# Performance of Artificial Intelligence–Powered ECG Analysis in Suspected ST-Segment Elevation Myocardial Infarction

**DOI:** 10.1016/j.jacadv.2026.102671

**Published:** 2026-03-20

**Authors:** Scott W. Sharkey, Robert Herman, Dawn R. Witt, Frank Aguirre, Mehmet Yildiz, David M. Larson, Avinash Murthy, Heather S. Rohm, Stephen W. Smith, Will Belzer, Jenny Chambers, Ellen Cravero, Seth Bergstedt, Greg Kerola, David Farmer, Andrew Willett, H. Pendell Meyers, Julia Harris, Christopher VanHove, Timothy D. Henry

**Affiliations:** a Minneapolis Heart Institute Foundation, Minneapolis, Minnesota, USA; b Cardiovascular Center Aalst, AZORG, Aalst, Belgium; c Powerful Medical, Bratislava, Slovakia; d Prairie Heart Institute at St. John’s Hospital, Springfield, Illinois, USA; e Carl and Edyth Lindner Center for Research and Education at The Christ Hospital, Cincinnati, Ohio, USA; f Department of Emergency Medicine, Hennepin County Medical Center and University of Minnesota, Minneapolis, Minnesota, USA; g Department of Emergency Medicine, Carolinas Medical Center and Wake Forest University School of Medicine, Charlotte, North Carolina, USA

**Keywords:** acute coronary syndrome, artificial intelligence, electrocardiography, percutaneous coronary intervention, ST-segment elevation myocardial infarction

## Abstract

**Background:**

Artificial intelligence (AI)–based electrocardiogram (ECG) analysis has emerged as a promising adjunct to human ECG interpretation in suspected ST-segment elevation myocardial infarction (STEMI).

**Objectives:**

To expand knowledge in this evolving field, the authors retrospectively analyzed the performance of a novel AI-ECG model in patients with cardiac catheterization laboratory activation for suspected STEMI.

**Methods:**

Consecutive patients were gathered from a multicenter U.S. STEMI registry (2018-2022) and categorized into 3 clinical cohorts based on the presence or absence of angiographic culprit and troponin elevation: acute myocardial infarction (AMI) with culprit, AMI without culprit, and no-AMI. Cardiac catheterization laboratory-activating ECGs were analyzed using an AI-ECG model trained to identify acute coronary occlusion and classified as occlusion myocardial infarction, OMI(+) or not, OMI(−).

**Results:**

The study included 2,523 patients, 68.3% male, with a median age of 63 years. AMI with culprit was present in 2076 (82.3%), AMI without culprit in 314 (12.4%), and no-AMI in 133 (5.3%). Among AMI with culprit patients, the model correctly identified 93.8% as OMI(+). Sensitivity for TIMI flow 0/1, 2, and 3 was 96.3%, 93.1%, and 86.9% respectively; *P* < 0.001. The model correctly identified 79.7% of no-AMI patients as OMI(−). The AUCROC was 0.952 (95% CI: 0.924-0.966). The AMI without culprit cohort included takotsubo syndrome OMI(+) = 78%, MI with nonobstructive coronary arteries OMI(+) = 61%, and myopericarditis OMI(+) = 67%.

**Conclusions:**

In suspected STEMI, this AI-ECG model correctly identified nearly all patients with acute coronary obstruction and most of those without AMI. If prospectively validated, this approach could improve management of patients with suspected AMI.

Emergency reperfusion is universally recognized as the definitive treatment for ST-segment elevation myocardial infarction (STEMI).^1^^,^^2^ This strategy is based on seminal research conducted more than 4 decades ago which established an anatomic link between electrocardiographic ST-segment elevation (STE) and acute coronary artery occlusion due to coronary thrombosis.^3^ Consequently, a treatment model has evolved whereby the electrocardiogram (ECG) is the “diagnostic gatekeeper” for identifying patients with acute coronary artery occlusion who require emergent reperfusion therapy.^4^ However, this approach has proven to be imperfect, since more than one-third of patients with angiographic acute coronary artery occlusion do not meet traditional electrocardiographic STE quantitative criteria.^5^, ^6^, ^7^ Furthermore, STE is present in a number of conditions other than acute coronary occlusion, including in normal individuals.^8^

When examining contemporary North American and European STEMI guidelines, it is evident that consensus is lacking regarding the most effective ECG criteria for emergent cardiac catheterization laboratory (CCL) activation in patients with suspected acute coronary artery occlusion (ie, STEMI).^9^ None of the numerous STEMI criteria have been prospectively validated against coronary angiographic findings or patient outcomes. The complexity and variability of these STEMI ECG criteria present a challenge for health care providers and can delay or deny timely reperfusion therapy for patients with an acute coronary artery occlusion. Furthermore, the emergence of the hospitalist care model in the U.S. has resulted in a scenario whereby patients with acute coronary artery occlusion, not recognized as having STEMI ECG changes at initial evaluation, may be labeled as non-STEMI with substantial delays to first cardiology evaluation and coronary angiography.

The aforementioned issues highlight the need to improve and standardize ECG interpretation with the goal of accurately identifying patients with acute total or subtotal coronary artery occlusion. Artificial intelligence (AI)–driven ECG interpretation, using deep neural networks trained and calibrated to angiographic findings in acute myocardial infarction (AMI) cohorts, has emerged as a promising tool to improve diagnostic accuracy while reducing variability in ECG interpretation, thereby leading to improved outcomes.^10^^,^^11^ To expand knowledge in this rapidly evolving field, we retrospectively analyzed the performance of a novel AI-ECG model among consecutive patients with emergent CCL activation for suspected STEMI in a large multicenter U.S. STEMI registry.

## Methods

### Patients

Patients were gathered from the Midwest STEMI Consortium from 2018 to 2022. This consortium includes 4 high-volume STEMI-percutaneous coronary intervention (PCI) centers: Minneapolis Heart Institute, Minneapolis, MN, Prairie Cardiovascular Institute, Springfield, IL, The Christ Hospital, Cincinnati, OH, and Iowa Heart Center, Des Moines, IA, although the Iowa Heart Center did not participate in the current study.^12^ The consortium registry is comprised of consecutive patients presenting with symptoms or signs of acute myocardial ischemia in whom the CCL was activated for suspected STEMI. CCL activation was typically initiated by an emergency department physician or trained emergency medical technician based on the clinical scenario together with the presence of electrocardiographic changes consistent with STEMI, STEMI equivalent (true posterior pattern, hyperacute T-waves, deWinter T-waves), or new or presumed new left bundle branch block (LBBB). All patients had emergent coronary angiography performed. CCL activations canceled by a cardiologist were not included in this study.

### Clinical definitions

AMI was defined as troponin elevation >99th percentile in the setting of acute myocardial ischemia. The culprit coronary artery and initial TIMI flow were determined by the interventional cardiologist performing the emergent coronary angiographic procedure. Based on coronary angiographic findings and troponin level, patients were placed into one of 3 cohorts: AMI with culprit = troponin >99th percentile *with* an angiographic culprit stenosis; AMI without culprit = troponin >99th percentile *without* an angiographic culprit stenosis (this cohort includes myocardial infarction with nonobstructive coronary arteries (MINOCA), takotsubo syndrome, and other conditions detailed in Results), and no AMI = troponin <99th percentile *without* an angiographic culprit stenosis.

### AI-ECG analysis

**Figure 1:**
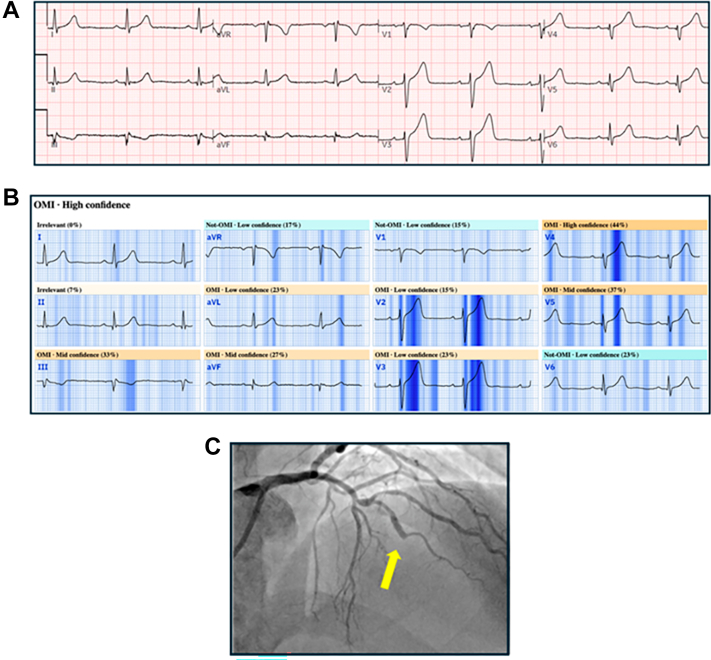
**Representative Example of Artificial Intelligence-Electrocardiogram Output and Corresponding Coronary Angiogram** (A). Electrocardiogram recorded in the emergency department. (B) Artificial intelligence-electrocardiogram output indicating presence of occlusion myocardial infarction. Darker blue = abnormal electrocardiogram regions identified by artificial intelligence model. (C) Coronary angiogram demonstrating complete occlusion of the mid-left anterior descending coronary artery (arrow). OMI = occlusion myocardial infarction.

All CCL-activating 12-lead ECGs were deidentified and retrospectively analyzed using the CE-certified PMcardio AI platform, which accepts raw waveform data or converts ECG images (PDF, PNG, JPEG) to digitized waveforms (retaining a sampling frequency of 320 Hz). The ECG digitization methodology has been described elsewhere.^13^ The primary deep neural network AI-ECG model (Queen of Hearts, eOMI v1, “Occlusive Myocardial Infarction Detected”) is calibrated to detect ECG signs of acute total or subtotal coronary artery occlusion (Figure 1). The model requires ≥2.5 seconds of waveform data per lead and produces a continuous probability score from 0 to 1. The AI-ECG model output is based solely on the ECG; no clinical data are considered. The training set definition of occlusive myocardial infarction (OMI) was: 1) angiographic acute culprit with TIMI flow grade 0/1/2; or 2) TIMIflow grade 3 with peak fourth-generation troponin T of at least 1.0 ng/mL or peak fourth-generation troponin I of at least 10 ng/mL.^7^ The U.S. multicenter registry cohort utilized in this study was entirely independent of the data sets used for model development and calibration, with no overlap in patients or institutions.

### Statistical methods

All statistical analyses were conducted using JMP 14.0 (SAS Institute). The AI-ECG model output is a continuous number between 0 and 1 indicating the probability of OMI. For binary performance analyses, the AI-ECG determined OMI (+) or OMI (−), was based on the previously validated threshold of 0.5 (on-label cut-off for the general chest pain population) and documented for each of the 3 clinical cohorts: AMI with culprit, AMI without culprit, and no AMI. Sensitivity was defined as the proportion of patients in the AMI with culprit cohort correctly identified as OMI (+) by the AI-ECG model. Specificity was defined as the proportion of patients in the no AMI cohort correctly identified as OMI (−) by the AI-ECG model. Positive predictive value was defined as the probability that patients with an AI-ECG output of OMI (+) were in the AMI with culprit cohort. Negative predictive value was defined as the probability that patients with an AI-ECG output of OMI (−) were in the no AMI cohort. The diagnostic performance of the AI ECG model was evaluated using a receiver-operating characteristic curve with calculation of the area under the curve (AUCROC). For these analyses, patients in the AMI without culprit cohort were excluded since this cohort includes diverse conditions such as MINOCA, takotsubo syndrome, and myopericarditis. The AI-ECG model was not trained to differentiate these conditions, and previous studies have failed to identify any unique ECG criteria which reliably separate these patients from the AMI with culprit patients.^14^^,^^15^ All ECGs in the AMI with culprit cohort with an AI-ECG model output of OMI (−), that is, AI-ECG false negatives, were retrospectively over-read by an experienced cardiologist (S.W. Sharkey) and categorized as to whether criteria for emergent CCL activation (ie, STE >1 mm in 2 contiguous leads or STEMI equivalent patterns) were present or absent.

Continuous variables with normal distribution are expressed as mean ± SD and compared using Student’s *t*-tests. Categorical variables are reported as frequencies (%) and compared with chi-square or Fisher exact tests. Confidence intervals (95% CIs) were calculated for each metric. All statistical tests were 2-sided with significance defined as a *P* value <0.05.

This study was approved by the Institutional Review Board at each of the participating institutions. The data sets supporting the findings reported in this study are available upon reasonable request to the corresponding author.

## Results

**Figure 2:**
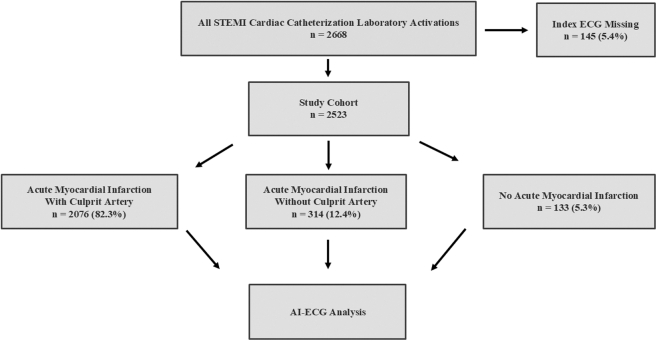
**Study Flow Diagram** AI = artificial intelligence; ECG = electrocardiogram; STEMI = ST-segment elevation myocardial infarction.

Between January 2018 and December 2022, the Midwest STEMI Consortium recorded 2,668 consecutive patients with CCL activation for suspected STEMI across 3 sites, of whom 145/2,668 (5.4%) were excluded from analysis because the activating ECG was missing. The remaining 2,523 patients included 2076/2,523 (82.3%) with AMI with culprit, 314/2,523 (12.4%) with AMI without culprit, and 133/2,523 (5.3%) with no AMI (Figure 2). The baseline characteristics of the 2,523 study cohort patients stratified by clinical cohort are shown in Table 1. For the entire cohort, 1723 (68.3%) patients were male, with a median age of 63 (IQR 55, 73) years, and 2,221 (88%) were Caucasian. An ambulance was used for transport to the emergency department in 935 (37%) of patients.

**Table 1:** Characteristics of 2,523 Consecutive Patients With Cardiac Catheterization Laboratory Activation for Suspected STEMI

	AMI With Culprit (n = 2,076, 82.3%)	AMI Without Culprit (n = 314, 12.4%)	No AMI (n = 133, 5.3%)	*P* Value
Age, y	64 (56, 73)	66 (54, 75)	58 (49, 69)	<0.001
Male	1,463 (70.5%)	167 (53%)	93 (70%)	<0.001
Race				0.007
African American	114 (5.5%)	18 (5.7%)	19 (14.3%)	
Asian	17 (0.8%)	1 (0.3%)	2 (1.5%)	
Caucasian	1,839 (88.6%)	280 (89.2%)	102 (76.7%)	
Hispanic	9 (0.4%)	2 (0.6%)	1 (0.8%)	
Not specified	97 (4.7%)	13 (4.1%)	9 (6.8%)	
Arrival transport				<0.001
Ambulance	785 (37.8%)	107 (34.1%)	43 (32.3%)	
Self/family	1,108 (53.4%)	151 (48.1%)	79 (59.4%)	
In-hospital	130 (6.3%)	45 (14.3%)	10 (7.5%)	
Not specified	53 (2.6%)	11 (3.5%)	1 (0.8%)	
Body mass index, kg/m^2^	29 (26, 33)	28 (23, 34)	29 (25, 33)	0.007
Diabetes	590 (28.4%)	79 (25.2%)	32 (24.1%)	0.30
Hypertension	1,296 (62.4%)	204 (65%)	83 (62.4%)	
Current smoker	667 (32.1%)	74 (23.6%)	30 (22.6%)	0.001
Prior AMI	439 (21.1%)	82 (26.2%)	27 (20.3%)	0.13
Prior PCI	514 (24.8%)	79 (25.2%)	25 (18.9%)	0.30
Prior CABG	133 (6.4%)	34 (10.8%)	10 (7.5%)	0.017
Initial HR, beats/min	79 (67, 95)	90 (77, 106)	86 (73, 98)	<0.001
LBBB	35 (1.7%)	18 (5.7%)	9 (6.8%)	0.017
Culprit coronary artery	728 (35.1%)	NA	NA	NA
LAD	827 (39.8%)			
RCA	260 (12.5%)			
CX	21 (1.0%)			
Diagonal	13 (0.6%)			
Obtuse marginal	16 (0.8%)			
Ramus intermedius PDA	15 (0.7%)			
Left main	28 (1.3%)			
Bypass graft	44 (2.1%)			
Other	124 (6%)			
Number of diseased vessels		NA	NA	NA
1	945 (45.6%)			
2	592 (28.5%)			
3	447 (21.5%)			
>3	32 (1.5%)			
Not specified	60 (2.9%)			
TIMI flow grade pre-PCI		NA	NA	NA
0/1	1,247 (60.1%)			
2	319 (15.4%)			
3	381 (18.4%)			
Not specified	129 (6.2%)			
PCI performed	1890 (91%)	NA	NA	NA
First medical contact to balloon time, min	108 (84, 142)	NA	NA	NA
CABG	64 (3.1%)	NA	NA	NA
Elective	16 (0.8%)			
Urgent	32 (1.5%)			
Not specified	16 (0.8%)			

AMI = acute myocardial infarction; CABG = coronary artery bypass grafting; CX = circumflex; HR = heart rate; LAD = left anterior descending; LBBB = left bundle branch block; NA = not applicable; PCI = percutaneous coronary intervention; PDA = posterior descending coronary artery; RCA = right coronary artery; STEMI = ST-segment elevation myocardial infarction.

### AMI with culprit

Among 2076 AMI with culprit patients, pre-PCI TIMI flow was 0/1 in 1,247 (60.1%); the culprit vessel was the left anterior descending (LAD) in 35.1%, circumflex (CX) in 12.5%, and right coronary artery (RCA) in 39.8% (Table 1). Single vessel coronary artery disease was present in 45.6% of patients and LBBB was present in 35 (1.7%) patients. Revascularization with PCI and/or coronary artery bypass grafting was performed in 1,954 (94.1%) patients. The median time from first medical contact to balloon inflation was 108 (IQR 84, 142) minutes. This time reflects the median of patients presenting directly to the primary PCI hospital together with those transferred from non-PCI sites.

### AMI without culprit

The 314 AMI without culprit patients were significantly older; *P* < 0.05, more commonly female; *P* < 0.001, with more frequent LBBB; *P* < 0.001 and less frequent current smoking status; *P* = 0.003, when compared to the AMI with culprit patients (Table 1). The clinical diagnostic categories for these patients are shown in Table 2. The 5 most common diagnoses, comprising 78.3% of the AMI without culprit cohort, were MINOCA, followed by takotsubo syndrome, ischemic cardiomyopathy, cardiac arrest, and myopericarditis.

**Table 2:** AMI Without Culprit Diagnostic Categories (N = 314)

Acute aortic dissection	1 (0.3%)
Acute neurologic event	2 (0.6%)
Acute pulmonary embolism	2 (0.6%)
Acute respiratory failure	9 (2.9%)
Cancer	2 (0.6%)
Cardiac arrest	28 (8.9%)
Cardiomyopathy ischemic	30 (9.6%)
Cardiomyopathy nonischemic	9 (2.9%)
Hypertensive emergency	6 (1.9%)
MINOCA	98 (31.2%)
Myopericarditis	27 (8.6%)
Sepsis	10 (3.2%)
Tachyarrhythmia	3 (1%)
Takotsubo syndrome	63 (20.0%)
Undetermined	24 (7.6%)

MINOCA = myocardial infarction with nonobstructive coronary arteries; other abbreviation as in Table 1.

### No AMI

The 133 no AMI patients comprised only 5.3% of the entire cohort. These patients were significantly younger; *P* < 0.001, more frequently African American; *P* < 0.001, with less frequent current smoking status; *P* = 0.02, and more frequent LBBB; *P* < 0.001 when compared to AMI with culprit patients (Table 1).

### AI-ECG performance

The AI-ECG model results for the AMI with culprit, the AMI without culprit and the no AMI cohorts are shown in Table 3.

**Table 3:** Performance of AI-ECG Model in 2,523 Consecutive Patients With Cardiac Catheterization Laboratory Activation for Suspected STEMI

	AMI With Culprit (n = 2,076)	AMI Without Culprit (n = 314)	No AMI (n = 133)	*P* Value
AI-ECG model OMI (+)	1948 (93.8%)	193 (61.5%)	27 (20.3%)	<0.001
AI-ECG model OMI (−)	128 (6.2%)	121 (38.5%)	106 (79.7%)	<0.001
AI model confidence level				
High	1933 (93%)	250 (79.6%)	104 (78.2%)	
Medium	85 (4.1%)	31 (9.9%)	17 (12.8%)	
Low	58 (2.8%)	33 (10.5%)	12 (9.0%)	
30-d mortality	124 (6.3%)	31 (10%)	5 (3.9%)	0.054

AI = artificial intelligence; ECG = electrocardiogram; other abbreviations as in Table 1.

#### AMI with culprit

**Figure 3:**
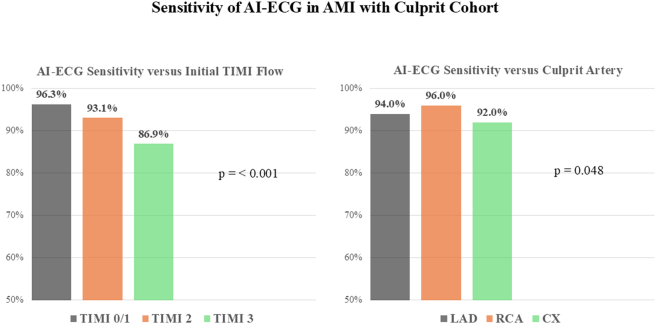
**Sensitivity of Artificial Intelligence-Electrocardiogram Model for Identifying Patients in the Acute Myocardial Infarction With Culprit Cohort versus Initial TIMI Flow and Culprit Coronary Artery** Sensitivity of artificial intelligence-electrocardiogram model for identifying patients in the acute myocardial infarction with culprit cohort vs initial TIMI flow (left) and culprit coronary artery (right). AMI = acute myocardial infarction; CX = circumflex; LAD = left anterior descending; RCA = right coronary artery; other abbreviations as in Figure 2.

Among the AMI with culprit patients, the overall sensitivity of the AI-ECG model was 93.8%. The sensitivity of the AI-ECG model for each category of initial TIMI flow and for each of the 3 major epicardial coronary arteries is shown in Figure 3. The AI-ECG sensitivity was greatest for TIMI flow grade 0/1 and the RCA and lowest for TIMI flow grade 3 and the CX coronary artery. The overall sensitivity for men vs women was 93.6% and 94.5%, respectively; *P* = 0.45. Among patients with LBBB, the AI-ECG model sensitivity was 20 of 35 (57.1%).

There were 128 AMI with culprit patients in whom the AI-ECG model output was OMI (−), representing model false negative interpretations. Among these patients, the initial angiographic flow was TIMI flow grade 0/1 in 46 (39%), TIMI flow grade 2 in 22 (19%), TIMI flow grade 3 in 50 (42%), and unknown in 10 (8%). Emergent PCI or CABG was performed in 109 (85%) patients. For the model false negative patients, the cardiologist retrospective ECG overread indicated a CCL activating pattern was present in 81 (63%) and absent in 47 (37%). In those with an activating pattern present, the ECG demonstrated STE in 57 (70%), a STEMI equivalent pattern (true posterior infarction, hyperacute T waves, de Winter T waves) in 4 (5%), and LBBB in 20 (25%). In the 47 patients without a CCL-activating pattern, the ECG demonstrated ischemia without STE (T-wave inversion, Q-wave, or ST-segment depression in 15 (32%), right bundle branch block conduction in 11 (23%), other (artifact, normal, ventricular arrhythmia) in 11 (23%), and minor STE in 10 (21%). If the 47 patients in whom STEMI or STEMI equivalent ECG criteria were absent are excluded from the analysis, the sensitivity of the AI-ECG model for AMI (+) culprit increased from 93.8% to 96.0%.

#### AMI without culprit

**Figure 4:**
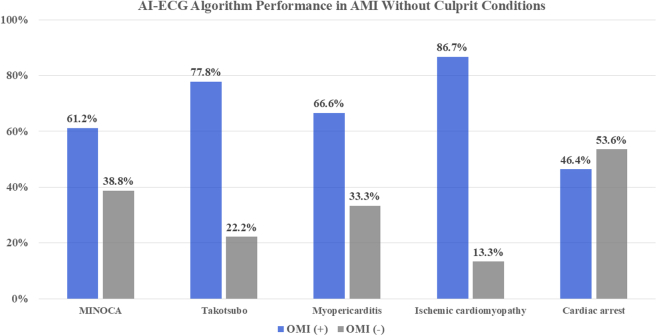
**Artificial Intelligence-Electrocardiogram Model Performance in the 5 Most Common Acute Myocardial Infarction Without Culprit Cohort Diagnostic Categories** MINOCA = myocardial infarction with nonobstructive coronary arteries; other abbreviations as in Figures 1 to 3.

The AI-ECG model performance for the 5 most common clinical diagnoses within the AMI without culprit cohort is shown in Figure 4. There was wide variability in the AI-ECG model output in these conditions. An OMI (+) output was most common (86.7%) in patients with ischemic cardiomyopathy and least common in those with cardiac arrest (46.4%).

#### No AMI category

Among the no AMI patient cohort, the AI-ECG model identified 106 of 133 (79.7%) patients as OMI (−) (Table 3).

#### Overall AI-ECG performance

The AI ECG model achieved an AUCROC of 0.952 (95% CI: 0.924-0.966), Central Illustration. The sensitivity for AMI with culprit was 93.8% (95% CI: 92.7%-94.8%) and the specificity for no AMI was 79.7% (95% CI: 71.9%-86.2%). The positive and negative predictive values were 98.6% (95% CI: 98%-99.1%) and 45.3% (95% CI: 38.8%-51.9%), respectively.

**Central Illustration:**
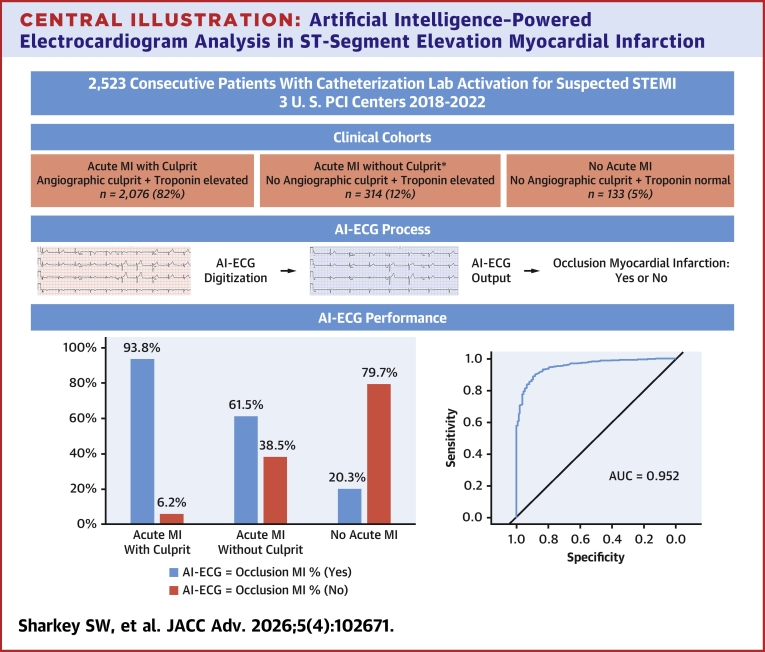
**Artificial Intelligence**–**Powered Electrocardiogram Analysis in ST-Segment Elevation Myocardial Infarction** In this retrospective analysis of ST-segment elevation myocardial infarction activations at 3 experienced U.S. percutaneous coronary intervention centers, the artificial intelligence-electrocardiogram model correctly identified 93.8% of patients with biomarker elevation and an angiographic culprit artery as occlusion myocardial infarction. The model correctly identified 79.7% of patients without either biomarker elevation or an angiographic culprit artery as no occlusion myocardial infarction. The receiver-operating curve demonstrates an area under the curve (AUCROC) of 0.952. ∗The acute myocardial infarction without culprit cohort represents unique conditions such as myocardial infarction with nonobstructive coronary arteries, takotsubo syndrome, and myopericarditis each with different artificial intelligence-electrocardiogram outputs. MI = myocardial infarction; PCI = percutaneous coronary intervention; other abbreviations as in Figure 2.

### Outcomes

The 30-day mortality for each patient cohort is shown in Table 3. Mortality was lowest (3.9%) in the no AMI cohort and highest (10%) in the AMI without culprit cohort, although the differences did not reach statistical significance; *P* = 0.054.

## Discussion

This study is among the first to examine the performance of a novel AI-ECG model among patients with CCL activation for suspected STEMI within a multiregional U.S. STEMI system. The principal findings include the following. 1) Among the AMI with culprit cohort, the overall sensitivity of the AI-ECG model was nearly 94%, performing equally well in men and women. Notably, the model sensitivity was greatest in patients with initial TIMI flow grade 0/1 (96%) and lowest in those with initial TIMI flow grade 3 (87%). Among patients with an angiographic culprit artery involving one of the 3 major coronary arteries, the model sensitivity was greatest for a RCA culprit (96%), followed by LAD culprit (94%) and CX culprit (92%). 2) The AI-ECG model correctly identified 80% of the no AMI cohort as OMI (−). 3) Among the AMI without culprit cohort, the AI-ECG model performance was quite variable, depending on the specific diagnosis.

We believe this multicenter STEMI registry is representative of experienced high-performing U.S. PCI centers. More than 80% of patients with CCL activation for suspected STEMI had troponin elevation with an angiographic culprit artery and the majority of these patients had TIMI flow grade 0/1 or 2 at initial coronary angiography. Moreover, only 5% of patients who proceeded to coronary angiography did not have biomarker elevation or an angiographic culprit, a scenario for which emergency CCL activation could be considered unnecessary.

### AMI with culprit

When considering the performance of the AI-ECG model, it is important to note that traditional STEMI CCL activation protocols are imperfect, failing to identify a substantial minority of patients with acute coronary artery occlusion.^5^ In fact, a recent study demonstrated that contemporary STEMI ECG criteria failed to identify nearly 40% of patients with an acute LAD occlusion.^16^ By default, these missed acute coronary occlusion patients are often classified as non-STEMI, therefore the true false negative rates of traditional STEMI CCL activation protocols are unknown.^17^

While the AI-ECG model used in this study performed well, an important minority (6%) were false negatives, of whom expert examination of the activating ECGs revealed the AI-ECG model did not recognize diagnostic ECG changes of STEMI in 65% (model failure). These false negative ECG patterns represent an opportunity for study, with the goal of improving model sensitivity. On the other hand, expert examination revealed the absence of currently accepted ECG STEMI criteria for CCL activation in 35% of these patients. In other words, the CCL was likely activated emergently for reasons other than the presence of ECG findings, for example, hemodynamic or rhythm instability, regional wall motion abnormality on point of care cardiac ultrasound, troponin elevation with ongoing chest pain, concern for left main coronary artery culprit, or resuscitated cardiac arrest. None of these variables were considered in the AI-ECG model and therefore the AI-ECG model should not necessarily be considered a failure in these circumstances.

New or presumably new LBBB in the presence of ischemic symptoms was a criteria for CCL activation at the hospitals participating in the current study. The AI-ECG model performance in LBBB was marginal, with a sensitivity in the AMI with culprit cohort of 57%. Furthermore, the prevalence of LBBB was lowest (1.7%) in the AMI with culprit cohort and greatest (6.8%) in the no AMI cohort, illustrating the lack of specificity of LBBB in identifying patients with acute coronary artery occlusion. The newly published 2025 American College of Cardiology/American Heart Association Joint Committee on Clinical Practice Guidelines for management of patients with acute coronary syndrome recognize the uncertainty regarding LBBB in acute coronary syndrome, stating “new or presumably new LBBB at presentation occurs infrequently and should not be considered diagnostic of AMI in isolation; clinical correlation is required.”.^1^ Whether the AI-ECG performance can be improved in LBBB requires further study.^18^^,^^19^

### AMI without culprit

This cohort includes several diverse conditions sharing common characteristics: symptoms of acute myocardial ischemia, electrocardiographic STE, troponin elevation, and absence of an angiographic culprit coronary artery stenosis. Advances in cardiovascular magnetic resonance imaging have allowed the identification of distinct conditions within this cohort, in particular MINOCA, takotsubo syndrome, and myopericarditis, each of which carry significant risk.^20^^,^^21^ These 3 conditions accounted for nearly 60% of all AMI without culprit cases. The AI-ECG model performance was inconsistent, yielding an OMI (+) diagnosis for MINOCA, takotsubo syndrome, and myopericarditis of 61%, 78%, and 67%, respectively. Whether an AI-ECG model can be trained to reliably recognize and separate these conditions is uncertain. In the meantime, coronary angiography is necessary to reliably distinguish these conditions from AMI with culprit.

### No AMI

Emergent CCL activation is usually unnecessary for patients without either troponin elevation or acute angiographic culprit coronary artery. The AI-ECG model correctly identified 80% of these patients, thus providing an opportunity to reduce CCL costs and lessen work-related stress for staff. In the current study, the no AMI cohort was small, representing only 5% of patients, likely reflecting the multidecade experience of the PCI centers with STEMI diagnosis and treatment. In the United States, the rate of unnecessary (false positive) CCL activations varies widely, reflecting different activation criteria, ECG interpretation experience, and population-level characteristics.^22^, ^23^, ^24^, ^25^, ^26^ Obviously, the AI-ECG model will have the greatest impact in those hospitals with high false positive activation rates.

### Study Limitations

This was a retrospective study limited to the Midwestern region of the United States and the majority of patients were Caucasian. In this study, only the CCL-activating ECG was analyzed, while in practice, the AI-ECG algorithm will usually be applied to the initial and subsequent ECGs. The performance of the AI-ECG model will undoubtedly vary depending on local experience with ECG interpretation and CCL activation criteria. This study was limited to patients with CCL activation for suspected STEMI and excluded those in whom the CCL activation was canceled before angiography (call offs). The performance of the model was not examined in patients with AMI in whom the CCL was not activated emergently, that is, those patients not meeting STEMI ECG criteria. These patients represent a large and clinically important cohort, a significant minority of whom have total coronary occlusion and requires further prospective study. Although, a core laboratory was not utilized to determine the angiographic culprit and initial TIMI flow, we believe our report is an accurate reflection of real-world experience. The high prevalence of patients in the AMI with culprit category may inflate the positive predictive value and may not reflect real-world experience. Finally, the CCL-activating ECG and the coronary angiogram were not simultaneous. It is well established that acute coronary thrombosis is dynamic, marked by episodes of spontaneous reperfusion.^27^^,^^28^ Therefore, ECG changes will likely reflect the status of the coronary artery at the time the ECG is recorded.

## Conclusions

This study demonstrates the potential for a novel AI-ECG model to rapidly and objectively identify patients with acute coronary artery obstruction and to exclude patients without AMI who do not require emergent CCL activation. This strategy can be implemented in any geographic location, including settings where local ECG interpretation skills and health care resources are limited, with the goal of providing rapid reperfusion therapy to a substantially larger number of patients and ultimately lowering AMI mortality.^29^ The clinical utility of this AI-ECG model requires prospective validation in diverse populations and clinical settings.

Perspectives **COMPETENCY IN MEDICAL KNOWLEDGE:** This retrospective multicenter U.S. STEMI registry demonstrated that AI-ECG interpretation correctly identified 94% of patients with a documented AMI and culprit coronary occlusion. The AI model sensitivity differed significantly among the 3 major coronary arteries: RCA (96%), LAD (94%), and CX (92%). The model sensitivity also differed significantly among TIMI flow grades: TIMI flow grade 0/1 (96.3%), TIMI flow grade 2 (93.1%), and TIMI flow grade 3 (86.9%). The model performance was variable in the cohort with AMI without culprit (eg, MINOCA, takotsubo syndrome, myopericarditis, and cardiac arrest. The AI model correctly identified 80% of patients without AMI (ie, those with unnecessary CCL activations).

**TRANSLATIONAL OUTLOOK:** For patients with suspected STEMI, an AI-ECG model is a promising tool with the potential to improve time to reperfusion while also reducing unnecessary catheterization laboratory activations. This technological advance can be integrated into diverse health care settings, offering a valuable assistive tool for providers regardless of their level of ECG interpretation expertise. Prospective implementation studies are necessary to evaluate whether integrating AI-based ECG interpretation in suspected AMI can shorten time to reperfusion and optimize resource utilization. Future studies should examine whether integration of clinical variables such as symptom duration, chest pain characteristics, and presence or absence of cardiac arrest might improve real-world applicability.

## Funding support and author disclosures

This study was funded by a Quality Initiative/Process Improvement Grant from the Accreditation Foundation Committee of the American College of Cardiology Foundation. Dr Herman is an employee of Powerful Medical and is the Chief Medical Officer and Co-founder of Powerful Medical. Drs Meyers and Smith serve as consultants to Powerful Medical and hold stock options. All other authors have reported that they have no relationships relevant to the contents of this paper to disclose.
